# Supplementary material for: Laser-Engraved Liquid Metal Circuit for Wearable Electronics
Source: Bioengineering (Basel). 2022 Jan 30;9(2):59. doi: 10.3390/bioengineering9020059 (PMC8869208; doi:10.3390/bioengineering9020059)
Supplement: Supplementary file 1 [file bioengineering-09-00059-s001.zip › bioengineering-1504560-supplementary.pdf]

Laser-Engraved Liquid Metal Circuit for Wearable Electronics

Shuting Liang [1,2,*], Xingyan Chen [1], Fengjiao Li [3] and Na Song [4]

Table S1. The relationship between carving speed, travel speed, carving time and engraving width in the sample.

Sample	Carving speed (mm/s)	travel speed (mm/s)	Carving time (s)	Engraving width (μm)
Sample 1	1.667	5	18	650
Sample 2	8.333	5	3	253
Sample 3	8.333	5	6	300
Sample 4	8.333	11.667	6	200
